# Multimodal Speaker Diarization Using a Pre-Trained Audio-Visual Synchronization Model

**DOI:** 10.3390/s19235163

**Published:** 2019-11-25

**Authors:** Rehan Ahmad, Syed Zubair, Hani Alquhayz, Allah Ditta

**Affiliations:** 1 Department of Electrical Engineering, International Islamic University, Islamabad 44000, Pakistan; 2 Analytics Camp, Islamabad 44000, Pakistan; s.zubair@analyticscamp.org; 3 Department of Computer Science and Information, College of Science in Zulfi, Majmaah University, Al-Majmaah 11952, Saudi Arabia; h.alquhayz@mu.edu.sa; 4 Division of Science & Technology, University of Education, Township, Lahore 54770, Pakistan; allahditta@ue.edu.pk

**Keywords:** speaker diarization, SyncNet, Gaussian mixture model, diarization error rate, speech activity detection, MFCC

## Abstract

Speaker diarization systems aim to find ‘who spoke when?’ in multi-speaker recordings. The dataset usually consists of meetings, TV/talk shows, telephone and multi-party interaction recordings. In this paper, we propose a novel multimodal speaker diarization technique, which finds the active speaker through audio-visual synchronization model for diarization. A pre-trained audio-visual synchronization model is used to find the synchronization between a visible person and the respective audio. For that purpose, short video segments comprised of face-only regions are acquired using a face detection technique and are then fed to the pre-trained model. This model is a two streamed network which matches audio frames with their respective visual input segments. On the basis of high confidence video segments inferred by the model, the respective audio frames are used to train Gaussian mixture model (GMM)-based clusters. This method helps in generating speaker specific clusters with high probability. We tested our approach on a popular subset of AMI meeting corpus consisting of 5.4 h of recordings for audio and 5.8 h of different set of multimodal recordings. A significant improvement is noticed with the proposed method in term of DER when compared to conventional and fully supervised audio based speaker diarization. The results of the proposed technique are very close to the complex state-of-the art multimodal diarization which shows significance of such simple yet effective technique.

## 1. Introduction

Speaker diarization systems assign speech segments to the people appearing in a dialogue. It is usually an unsupervised technique where the number of speakers is unknown and limited information is available about them. Its applications lay in broadcast news, TV/talk shows, meeting recordings, telephone recordings, etc. Diarization tasks are very challenging when unimodal data is available. Audio-based diarization has lot of complexities due to overlapping speech utterances from various speakers, environmental noise, short utterances and reverberations. Similarly, in video data, speakers may not face the camera, move in a multi-party interaction way or they can be occluded by other speakers. The use of the video modality limits one to using lip and face movement detection for diarization. For each available dataset, the configuration of the recording equipment also varies a lot. For example, audio data may be acquired from a far-field microphone array, individual lapel microphones/headsets or single omnidirectional microphones. Similarly, video recordings comprise of individual speaker closeup cameras, cameras covering some group of speakers or a wide camera covering all the available speakers in an event.

Conventional audio-based diarization [1,2,3,4,5,6,7,8,9,10] systems comprise of agglomerative hierarchical clustering (AHC) based on Hidden Markov model (HMM)/Gaussian mixture model (GMM) with Bayesian information criteria (BIC) [11] as a metric for merging clusters. This technique initializes a large number of HMM/GMM states e.g., K = 16, where each state represents one cluster/speaker. The complete audio is initially divided into K segments and each cluster is trained with one segment. For each cluster, BIC is computed and similar clusters are merged together based on the differences in BIC metric. This merging process works iteratively until no two clusters have a difference in BIC close to zero. With such a technique, the number of speakers and their temporal segments are acquired. In the above discussed audio-based approaches, Mel-frequency cepstral coefficients (MFCC) [12] features have been widely used. The advent of i-vector [13] feature embedding based on factor analysis [14] proved significant improvement in speaker verification and these embeddings were used in speaker diarization [15,16,17] problems, which provided significant results. Later, researchers started working on neural network-based embedding approaches which resulted in the d-vector [18], x-vectors [19] and other neural network embeddings [20,21]. In contrast to unsupervised diarization approaches, some fully supervised diarization [22,23] techniques were also proposed.

To solve the shortcomings and limitations in audio-based diarization approaches, multimodal approaches comprised of audio and visual modalities were proposed. Multimodal approaches either use active speaker detection using lip and face movements [24,25,26,27] or some audio-visual fusion technique [26,28,29] at the feature or output stage after applying diarization on individual modalities. The audio and visual modalities provide complementary information, so they are more likely to be robust as compared to audio-only or video-only diarization techniques. In the last decade, several multimodal diarization techniques have been proposed, e.g., [25,27,29,30,31,32,33]. The scenario of available recordings varies depending on participant speech turns, silence between speech utterances of different speakers, short speech utterances, overlapping speech and environmental noise. Moreover, the participants may be seated/static or move around.

In recently published multimodal diarization approaches, e.g., [32,33], the focus of the authors is to track active speakers based on speech source localization (SSL). In such approaches, SSL along with video domain processing makes the diarization process computationally intensive. Our proposed technique is comparatively simple and heavily inspired by the work of automatic lip syncing in the wild [34]. In this technique, authors are trained an audio-visual convolutional neural network to learn speech and mouth synchronization. The trained model is applicable to determine lip synchronization errors, active speaker detection and lip reading. We used the pre-trained model referred to as SyncNet, to find active speakers in short video segments of closeup camera streams. The focus of our work is to robustly identify active speakers using the pre-trained SyncNet model. Our introduced diarization approach comprises acquiring features in the audio domain and applying GMM-based clustering on those audio frames which were robustly identified as corresponding to an active speaker. The simplicity of the approach is also reflected in the video domain, where we apply face detection, convert the results into short video segments and feed them to an audio-visual pre-trained model to apply inference. Compared to the audio-based diarization techniques which consist of conventional and fully supervised diarization, our results are very significant and prove the validity of such a novel approach. Compared to one of the complex multimodal techniques our approach provides nearly similar results.

The rest of the paper is divided as follows: Section 2 provides a detailed literature review. In Section 3 we describe each part of the proposed technique which consists of audio and video preprocessing, the SyncNet architecture details and a complete diarization pipeline. Section 4 comprises experimentation details which describe the audio-visual dataset, the parameters used in each audio and video pipelines, comparison methods, evaluation metrics, environmental setup and finally results and discussion. In Section 5, we provide concise conclusions regarding our work and results.

## 2. Literature Review

Speaker diarization requires grouping homogeneous speaker regions when multiple speakers are present in any recording. This task is usually performed with no prior knowledge about speaker voices or their number. The speaker diarization pipeline consists of audio feature extraction where MFCC is usually a choice for representation. Speech activity detection is then applied to separate speech and non-speech MFCC frames followed by segmentation and clustering techniques which results in final diarization. Use of the video modality is motivated by fact that audio and video have correlated factors. For example, the lip, face and head movement of an active speaker are highly correlated with his speech. Hence, features extracted from frontal views of speaker faces can be used to discriminate the active speaker. Such visual features are used in speech recognition [35,36], speech source separation [37,38] and speaker diarization [39,40,41,42]. 

Friedland et al. [43] proposed the use of compressed domain video features for multimodal speaker diarization that comprises frame-based visual activity features. These features were computed as a motion vector magnitude. Multimodal fusion was applied for MFCC and video features by a weighted likelihood of Gaussian mixture model. An agglomerative hierarchical clustering technique was used where each cluster was modelled by joint audio and video GMM. In contrast to its simplicity and less computational complexity, this technique might not work in scenarios where speakers move from their position or silent speakers shake their heads while listening. In [44], Garau et al. provided a comparison of two audio-visual synchronization methods. These two methods consist of canonical correlation analysis (CCA) and mutual information (MI) which uses MFCC features along with motion features from face tracks. The MI performed slightly better than CCA. Moreover, it was concluded that lip and chin vertical-movement visual features correlate the most with speech. Similarly, mutual information, which combines acoustic energy and gray-scale pixel’s value variation, was also used by Noulas et al. [30]. A dynamic Bayesian network was used to jointly model the audio and visual features for speaker diarization. Experiments were conducted on meeting recordings consisting of four speakers who face the camera and broadcast news with five people, where only three of them speak. Later, El Khoury et al. [29] proposed audiovisual diarization of people, where individual audio and visual clustering is carried out and fused together using co-occurrence matrices. The audio pipeline consists of MFCC feature extraction followed by SAD and finally segmentation and clustering. Similarly, in the video domain initially shot detection is applied then face detection, people tracking, people clustering and finally face clustering. Audiovisual diarization finally combines both clusters using an association technique. Minotto et al. [25] solved speaker diarization problems through speech source localization (SSL) in the audio domain and face detection and tracking in the video domain. A final decision is made using a supervised support vector machine (SVM) classifier. SSL provides lot of advantage in the analysis because recordings of two or three speakers consist of lots of overlapping speech segments. In [27] Sarafianos et al. applied audio-visual diarization using Fisher linear semi-discriminant analysis. After individual audio and video diarization, audio-visual fusion is applied. Kapsouras et al. [31] proposed to cluster face features and audio features independently and then correlate them based on temporal alignment. The most recent works in diarization [32,33] mainly focus on the use of the sound source localization (SSL) technique to find active speakers. This technique helps to robustly identify speech overlap regions. Cabañas-Molero et al. [32] proposed to use SSL in the audio domain and motion measurements along with lip movement in the video domain. Both domains are fused together via a decision algorithm. The localization algorithm is evaluated on space volume rather than a discrete point in the space. Similarly, Gebru et al. [33] proposed multimodal speaker diarization based on spatiotemporal Bayesian fusion, where a supervised localization technique is used to map audio features onto the image. This is achieved by sound source localization in the audio and multiple person visual tracking in the video which are fused via a supervised technique.

In most of the techniques discussed above, either a source localization technique is applied in audio pipeline to locate the active speaker or audio clustering/diarization is applied separately. Similarly, in the video domain face tracking, mouth/lip movement, motion measuring techniques are applied to get diarization results. Finally, audiovisual fusion is applied on the feature level or output level. Both audio and video pipelines require excessive processing to acquire the individual and fusion results. Comparatively, our technique is simple and relies more on a pre-trained SyncNet model to find active speakers. A simple preprocessing pipelines in the audio and video domain is required, which finally ends up in audio-based clustering to acquire diarization. This technique is well suited for formal meeting scenarios where people are static/seated and frontal faces are captured most of the time.

## 3. Methodology

**Figure 1:**
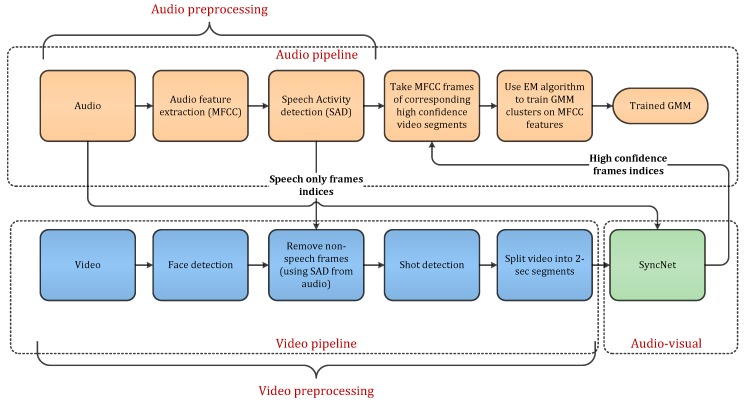
Multimodal speaker diarization pipeline with pre-trained audio-visual synchronization model.

In this section we describe each part of our proposed method in detail. First of all, from Figure 1, we describe the audio and video preprocessing pipelines, the SyncNet architecture and its inference on short video segments. Afterwards, we describe our complete diarization pipeline which consists of the sequence of steps involved in applying complete diarization.

### 3.1. Audio Preprocessing

For audio part we use a mix-headset audio recording that consists of voices from all the speakers. We extract the Mel-Frequency Cepstral Coefficients (MFCC) [12] features and normalize it by zero mean and unit variance. Then we apply energy-based speech activity detection (SAD) to classify speech and non-speech frames. For that purpose, we use available annotations and make non-speech audio samples equal to zero. In the SAD block, MFCC features were concatenated with energy features. A support vector machine classifier is applied to classify speech and non-speech frames which is trained on the 10% highest and 10% lowest energy frames. The SAD block provides speech only in MFCC frames and we discard non-speech frames. Such optimal SAD is actually applied to focus on the diarization technique and its subsequent improvement. Figure 1 shows the audio preprocessing pipeline consisting of MFCC feature extraction and SAD.

### 3.2. Video Preprocessing

**Figure 2:**
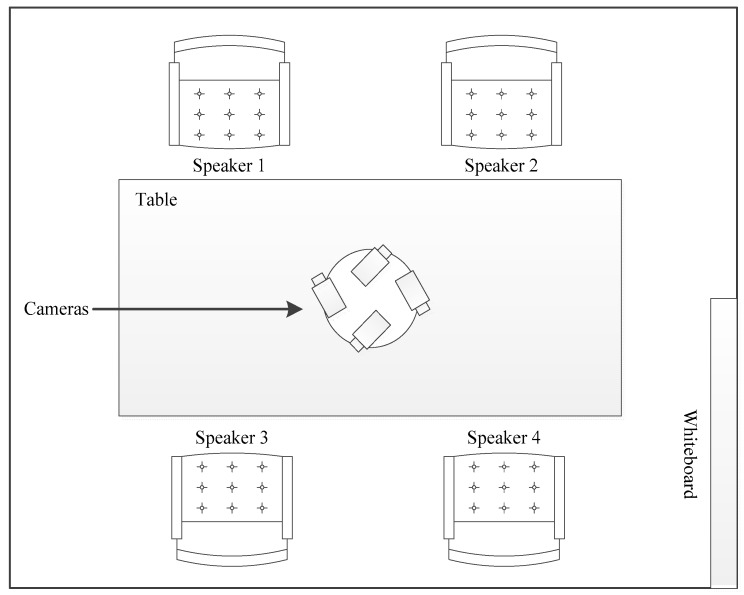
Representation of the AMI meeting room.

From the Augmented Multi-party Interaction (AMI) [45] corpus, the available video dataset consists of multiple recordings from cameras mounted in different room places. To capture the face of an individual speaker, we use a closeup camera mounted on the tabletop. This camera configuration is presented in Figure 2, where four tabletop cameras are mounted to capture the individual speakers. Face detection is applied on each closeup camera stream and the face-only region is extracted. Afterwards, video frames consisting of silent parts are removed using audio SAD’s speech-only frame indices. The shot detection technique is then applied to track continuous frames which contain faces and split the video frames into each shot where the face detector misses its detection. As in audio-based diarization, segment length is usually defined based on the assumption that each speaker will speak for at least a particular segment time. In conventional audio speaker diarization techniques based on HMM/GMM, each cluster is trained on speech frames consisting of at least one segment length duration. In the video part, 2-second segment length is selected to split the video shots into smaller video segments. This can help to identify active speakers in each short video. For each video segment, audio-visual synchronization is determined between the audio and mouth motion in a video. For that purpose, we utilized a pre-trained SyncNet model which finds how much audio belongs to the visible speaker.

### 3.3. SyncNet Architecture and Inference

The SyncNet architecture was proposed in [34], where the authors developed a two-stream model consisting of an audio-visual convolutional neural network with contrastive loss. The model was trained on several hundred hours of speech from BBC videos that include hundreds of speakers. Audio data with a sampling frequency of 16 KHz is converted into 13-MFCC features at the rate of 100 Hz. The audio part of the network is provided with 0.2 s of speech consisting of 20 MFCC frames, hence a 13 × 20 dimensional input. The input to the visual part of the network is the face region which is extracted using a face detection technique. For a 25 Hz video frame rate, five consecutive video frames are used, which gives 0.2 s of video segment. For the video network, the input data is of 120 × 120 × 5 dimension. The SyncNet architecture takes the output of both the audio and video for the last fully connected layer and applies contrastive loss to minimize the distance between genuine audio and corresponding video pairs. This loss is described as follows:

(1) $$E=\frac{1}{2N}\overset{N}{\underset{n=1}{\sum }}\left(y_{n}\right)d_{n}^{2}+\left(1-y_{n}\right)\max{\left(margin-d_{n},0\right)}^{2}$$

(2) $$d_{n}=||v_{n}-a_{n}||{}_{2}$$

where a and v are the outputs of the last fully connected layers, y∈[0,1] is the binary similarity metric between the input and video inputs.

In our proposed work, we used a pretrained SyncNet model to determine the the active speaker in each closeup video. As discussed in video preprocessing section, we split the video shots into 2 s segments and then apply SyncNet inference on each video segment. The SyncNet model outputs two metric values: offset and confidence, which are computed to determine the audio-visual relationship. Segments which have lowest offset and high confidence values determine that the visible speaker is the one who is speaking. In our approach, we define two threshold values based on the analysis of those short segments in which complete audio segment belongs to the visible speaker. The first one is the offset threshold, which is defined as $Th_{of}=\left[0,t_{1}\right]$, which only selects those video segments whose audio-visual offset value is between 0 and $t_{1}$ (both inclusive). After shortlisting the video segments by applying the first threshold we apply the second one, that is the confidence threshold $Th_{conf}>t_{2}$. It only selects those video segments whose audio-visual matching confidence is greater than $t_{2}$. These two types of thresholds hierarchically select only those video segments whose audio matches the visible speaker with high confidence. Thus, we call them high confidence video segments. The video frame indices of high confidence video segments provided by SyncNet are used in the audio pipeline to train a GMM cluster on the corresponding MFCC frames. Finally, for each closeup video which belongs to one speaker we train one GMM. We call such a cluster a pure GMM because it is only trained on high confidence frames.

### 3.4. Complete Multimodal Diarization Pipeline

Audio preprocessing pipeline provides speech-only MFCC frames after applying speech activity detector and video preprocessing pipeline-provided 2 s video segments. After acquiring the inference results from the SyncNet architecture we used high confidence segments only and take MFCC frames of those corresponding segments. We train a GMM model using Expectation Maximization (EM) with MFCC frames, so for each closeup video a GMM model with K mixtures is trained on the corresponding high confidence MFCC frames only. We represent a mixture of Gaussians as follows:

(3) $$p\left(x|\mu ,\Sigma \right)=\overset{K}{\underset{i=0}{\sum }}\pi_{i}N\left(x,\mu_{i},\;\Sigma_{i}\right)$$

where $\mu_{i}$ represents the means vector for each mixture, $\pi_{i}$ is the mixture coefficient and $\Sigma_{i}$ is the covariance matrix. In expectation step responsibilities are calculated as follows:

(4) $$r_{jc}=\frac{\pi_{c}N\left(x_{j}|\mu_{c},\;\Sigma_{c}\right)}{\sum_{i=0}^{K}\pi_{i}N(x_{i}|\mu_{i},\;\Sigma_{i})}$$

While calculating $r_{jc}$, j represents the $j^{th}$ datapoint and c represents the mixture number. The maximization step consists of calculating mean vectors, covariance matrix and mixture components as follows:

(5) $$\mu_{c}^{new}=1\backslash N_{c}\underset{j}{\sum }r_{jc}x_{j}$$

(6) $$N_{c}=\underset{j}{\sum }r_{jc}$$

(7) $$\Sigma_{c}^{new}=\frac{1}{N_{c}}\underset{j}{\sum }r_{jc}\left(x_{j}-\mu_{c}^{new}\right){\left(x_{j}-\mu_{c}^{new}\right)}^{T}$$

(8) $$\pi_{c}=\frac{N_{c}}{n}$$

where n is the total number of data points in the data set.

Now, such a cluster is assumed to be pure and it is most likely to be trained on one speaker. After all the clusters have been trained, which are now speaker-dependent clusters, the likelihood of the rest of the MFCC frames from each cluster is computed. The most likely frames are assigned to that specific cluster. Finally, all the frames are assigned to one of the clusters and the diarization error rate is computed.

In speaker diarization problems the number of speakers is usually unknown, so we assume the number of speakers equals to the number of available closeup videos. In such scenarios where only one recording is available, one can assume the number of speakers equals the number of distinct faces in all the video frames. Our diarization technique is designed for such scenarios where all the speaker faces are visible in the video. Additionally, we provided the code of proposed multimodal diarization at [46].

## 4. Experimentation Details

### 4.1. AMI Meeting Corpus

**Figure 3:**
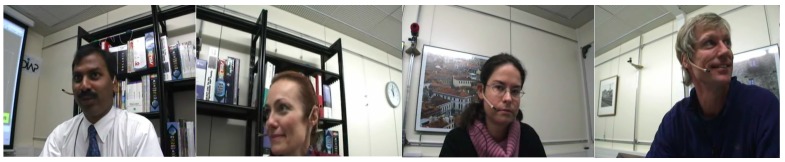
AMI IS1008a closeup camera images of individual speakers.

We tested our proposed method on a popular subset of the AMI [45,47] meeting corpus that consists of 5.4 h of recordings for the audio and 5.8 h of multimodal recordings. These meetings were recorded in English with mostly non-native speakers. The acoustic properties of the recordings also vary due to the different room scenarios. For the audio dataset we used a mix-headset recording which contains voices from all the speakers. In this dataset, the video recordings consist of individual speaker and room view cameras. In our work, we used the individual speaker cameras which are also known as closeup cameras. All the recordings consist of four speakers and each recording is labeled with and id and the lower-case letters a-c, which indicate the session of that particular recording. Our used subset is taken from the Idiap scenario meetings (IS) set. Figure 3 shows some closeup camera samples from meeting IS1008a. Each session consists of 15-35 min of recording.

### 4.2. Audio Pipeline

In the audio pipeline we extract 19-dimensional MFCC features with window length of 30 ms and hop length of 10 ms. MFCC frames thus have sample rate of 100 Hz. In the SAD block, MFCC features along with energy features are used with the same window and hop length. An SVM-based classifier is used to train speech and non-speech frames. Further, when the audio pipeline gets high confidence video segment information, a GMM with 20 components and diagonal covariance matrix is trained using an Expectation Maximization algorithm. For each closeup video, the number of high confidence video segments varies, which leads to different numbers of MFCC frames for each speaker. The specification of the GMM model for each speaker is kept same, i.e., 20 components, diagonal covariance.

### 4.3. Video Pipeline and SyncNet

Given each closeup video with a frame rate of 25 fps, we apply face detection with subsequent SAD and shot detection. All the available shots are then further segmented into 2 s chunks to apply SyncNet. This segment length is selected based on the assumption that each speaker may speak for a minimum of 2 s. Moreover, shots and segments smaller than seven frames are also discarded because they are too small to provide reliable audio-visual matching. As two metrics are computed on the output of SyncNet’s inference that is offset and confidence, two threshold values were applied to weed out unsynchronized audio-visual segments. To select high confidence video segments, we choose an offset threshold range between 0 and $t_{1}$, where $t_{1}=3$. Secondly, confidence threshold is applied with a value $t_{2}=1.5$. Segments with offsets of less than 0 and greater than 3 are discarded. Similarly, if the offset value is within the threshold then segments with confidence less than 1.5 are also discarded. After applying these two threshold values and discarding the video segments, it is more likely that in the remaining video segments complete audio only belong to the visible speaker.

### 4.4. Evaluation Metric

The evaluation metric for speaker diarization is the Diarization Error Rate (DER). It is a combination of four errors: False alarm ($E_{FA}$), Missed speech ($E_{MS}$), Speaker error ($E_{SE}$) and Overlapping speech error ($E_{OS}$). $E_{FA}$ is defined as a fraction of time where non-speech is hypothetically labelled as speech. $E_{MS}$ is the fraction of time when actual speech is labeled as a non-speech in the hypothesis. These two errors directly belong to the speech activity detector. The other two errors are $E_{SE}$ when the wrong speaker is assigned and $E_{OS}$ when the reference has multiple speakers and it is not labelled as such in the hypothesis. Finally, DER is sum of all these errors, defined as follows:

$$DER=E_{FA}+E_{MS}+E_{SE}+E_{OS}$$

### 4.5. Comparison Methods

To compare the proposed multimodal technique, first we use the conventional speaker diarization (SD) consisting of agglomerative hierarchical clustering [5] based on HMM/GMM. Such a technique initializes with a large number of clusters, e.g., 16, and then hierarchically merges them based on a Bayesian information criterion [11], which eventually ends up giving an optimal number of clusters (speakers). The first method to our comparison is conventional speaker diarization (SD) [9] based on GMM-based hierarchical clustering which is a completely unsupervised technique. Our second comparison method is fully supervised speaker diarization (SD) described in [23] which employs speech activity detection and speaker change detection [48] based on bidirectional LSTM [49], neural speaker embedding [50] based on a LSTM network and triple loss function. All these modules are combined in the speaker diarization pipeline and are jointly optimized with affinity propagation [51] clustering. Each module of this method is fully supervised and trained on about 70% of the AMI meeting corpus, while our proposed method is completely unsupervised. The subset of the AMI corpus used in our approach is either part of the training or development set in this competing method, which makes this comparison very challenging for the proposed approach. The Results section describes this in detail.

Thirdly, for the completeness of this research a comparison to the state-of-the art multimodal technique is carried out. This multimodal technique is described in [32] where the authors used sound a source localization (SSL) technique in the audio domain and motion & lip movement measures in the video domain. The SSL technique is used to detect active speakers and overlapping speech detection in the audio domain. Finally, the outputs from both streams are combined through a decision algorithm to acquires the diarization results. We compared our results with particular scenarios where the speakers are seated and do not stand up and move towards a whiteboard or the screen.

Comparison to other multimodal techniques are difficult since the scenarios of the recordings and proposed techniques in each of them varies significantly. Specifically, important factors that motivate one to develop any diarization technique vary depending on overlapping speech intervals, the recording equipment used in term of cameras and mic arrays, available speaker information and available training data. Some of the recent multimodal diarization techniques [25,33,52] employ sound source localization techniques in audio pipelines along with motion detection, face detection or mouth/lip movement and finally audio-visual fusion in the video pipeline. The techniques proposed in these papers are heavily oriented towards the sound source localization and data sets used such as AVDIAR [33], MVAD [25] and AVASM [53] contain large fractions of overlapping speech. However, our proposed technique doesn’t employ any localization technique in the audio pipeline, but rather it locates active speakers through audio-visual synchronization. Secondly, we didn’t consider determining overlap speech regions, although overlapping speech error is included in computing the diarization error rate.

### 4.6. Environmental Setup

We developed our audio-visual speaker diarization system in Python using Anaconda Python distribution on the Windows 10 platform on a computer equipped with a 2.6 GHz CPU, 16 GB RAM and a 3 GB Nvidia GTX 1060 GPU. Librosa [54] is used for audio feature extraction. For face detection, we used the face_recognition method given at https://github.com/ageitgey/face_recognition, which is based on dlib [55]. Pyannote.metrics [56] is used to extract the DER.

### 4.7. Computational Cost

In the propose system depicted in Figure 1, majority of the cost comes from the video pipeline because audio pipeline consists of MFCC feature extraction, SAD’s inference and computing GMM’s likelihood. These processes are very quick and requires fewer computations. In video pipeline, face detection is based on dlib’s [55] convolutional neural network (CNN). The computation time of this face detection module with image size of 288×352 on Nvidia GPU GTX1060 is about 0.03 s. With such resolution this module can process approximately 33 frames per second which is quicker than the frame rate of available recordings which is 25 fps. The processing time of the face detector increases linearly with the size of the image. Given a number of pixels N=101,376=288×352, the frame rate is F=33. If the number of pixels is scaled by an integer number s that is N∗s, then the frame rate would be F/s. The computation time of each frame for four camera streams would be:

$$C_{t}=4\;s/F$$

Next, the computational complex block is SyncNet which is a two streamed (audio and visual) Convolutional neural network. The input to this block is five frames of size 120×120 each. Each short video segment in our case comprises of maximum 2 s length which is provided to SyncNet for inference. This module processes each short segment in approximately 0.6 s. The total complexity of this module depends on the number of short video segments. This number varies for each camera recording. Computation time of this module for each camera is:

$$C_{sync}=0.6\;v_{s}$$

where $v_{s}$ is the total number of short video segments.

### 4.8. Results and Discussion

Table 1 presents the diarization error rate in term of percentage for the proposed and the first comparison method based on agglomerative hierarchical clustering (AHC) discussed in [9]. The table shows DER values for individual recordings, their difference in terms of improvement, average DER of all the recordings and finally average improvement. Any negative value in improvement column indicates a reduction in performance. The conventional method is a completely unsupervised technique where the actual number of speakers are unknown. The recording scenarios of the meeting corpus used in the comparison vary significantly in terms of overlapping speech duration, speaker movements towards the whiteboard and short utterances. In all the recordings we noticed a significant improvement and the maximum error reduction noticed in IS1003b is about 29.5%. Moreover, on the average results for the whole subset, a 13.6% error reduction is achieved. This is due to the fact that the proposed technique creates pure audio clusters with the help of high confidence video frames acquired from the audio-visual SyncNet model. Such a technique significantly reduces the speaker error which assigns wrong speakers to the audio segments.

The second comparison to our method is with a fully supervised speaker diarization technique [23] which is very challenging to the proposed methodology. One of the recordings, IS1003b, is not included in the comparison because it was not part of any training, testing or development set. Table 2 shows the DER for individual recordings with their gain or reduction in improvement. The third column represents that the recording is either part of the training (Train) or development (Dev) set. In [23], development set recordings are used for hyperparameter optimization for the speech activity detector, speaker change detector and speaker diarization pipelines. When compared with the proposed method we noticed a DER improvement in most of the recordings, while, in the training subset we see both increases and decreases in DER. A noticeable improvement from the training set is gained for the IS1000a recording while the maximum impairment is seen for IS1006b. Overall, the average improvement is 1.4% for the proposed multimodal method. The maximum improvement gain is 17.2% for IS1000a.

Finally, Table 3 presents the results of a multimodal technique where 5.8 h of a particular subset of IS recordings are taken in which all the speakers are seated. The reason for choosing this subset which comprises of static/seated speakers is that the proposed technique does not employ any localization technique. The results clearly show that our proposed technique performs nearly same to the state-of-the art multimodal approach with just 0.56% impairment. It shows that such a technique is as effective as any such complex diarization approach.

## 5. Conclusions

In this paper we have presented multimodal diarization technique using a pre-trained SyncNet model. In the audio pipeline we apply feature extraction, speech activity detection and finally a clustering technique for speaker diarization. Only high confidence MFCC frames acquired through SyncNet inference are used to train GMM models and then the rest of the audio frames are clustered on the basis of maximum likelihood. In the video pipeline, we apply face detection to crop face-only regions, remove silence frames, shot detection and finally split the video into 2 s short segments. These short video segments are then provided to pre-trained SyncNet model which runs the inference on them. On the output results of this inference we apply two thresholds to select those video segments which are confident enough on the active speaker in the video and its respective audio domain.

When compared with audio-based diarization techniques we noticed the effectiveness of such a novel multimodal diarization. The main advantage of our proposed method is in providing pure clusters which are trained on frames belonging to a single speaker, while in the conventional diarization approach which uses an agglomerative hierarchical clustering technique, there is greater chance of impurity in term of merging clusters that have voices of multiple speakers. Our technique, that is based on the threshold selections for offset and confidence metrics in the video domain, robustly prevents any such contamination. Beside this, our proposed technique is fully unsupervised and doesn’t even require any out of domain data for training purposes. Furthermore, the proposed technique is applicable to meeting recordings, TV/talk shows and movies where speakers face the camera most of the time. Finally, it is also concluded that such technique is similar in accuracy to one of the compared multimodal approaches.

In the future, we plan to incorporate overlapping speech detection in the video pipeline, where multiple speakers speak simultaneously. For that purpose, a model similar to SyncNet would be explored to label overlapping speech segments. Secondly, sound source localization techniques will also be explored to detect active speakers in scenarios where users move or do not face the camera.

## Figures and Tables

**Table 1:** Comparison of diarization error rate (%) score with conventional speaker diarization (SD).

Meeting ID	Conventional SD [9]	Proposed Multimodal	Difference (Improvement)
IS1000a	42.079	29.313	12.766
IS1001a	42.144	37.573	4.571
IS1001b	48.301	35.709	12.592
IS1001c	52.889	24.389	28.5
IS1003b	51.681	22.169	29.512
IS1003d	68.443	48.655	19.788
IS1006b	52.65	42.861	9.789
IS1006d	66.849	58.497	8.352
IS1008a	16.172	10.946	5.226
IS1008b	12.075	12.715	-0.64
IS1008c	40.59	22.217	18.373
IS1008d	35.503	21.376	14.127
**Average**	44.11	30.535	-
**Average Improvement**	13.58		

**Table 2:** Comparison of diarization error rate (%) score with a fully supervised speaker diarization (SD) system.

Meeting ID	Fully Supervised SD [23]	Recording Set	Proposed Multimodal	Difference (Improvement)
IS1000a	46.55	Train	29.313	17.237
IS1001a	43.31	Train	37.573	5.737
IS1001b	26.77	Train	35.709	-8.939
IS1001c	25.74	Train	24.389	1.351
IS1003d	59.56	Train	48.655	10.905
IS1006b	29.87	Train	42.861	-12.991
IS1006d	51.06	Train	58.497	-7.437
IS1008a	13.84	Dev	10.946	2.894
IS1008b	14.97	Dev	12.715	2.255
IS1008c	22.26	Dev	22.217	0.043
IS1008d	26.25	Dev	21.376	4.874
**Average**	32.74		31.29	-
**Average Improvement**	1.44			

**Table 3:** Comparison of diarization error rate (%) score with a multimodal speaker diarization (MMSD) system.

Meeting ID	MMSD [32]	Proposed Multimodal	Difference (Improvement)
IS	21.68	22.24	−0.56
